# The prognostic role of frailty on outcomes following meningioma resection in the elderly: a matched analysis employing baseline functional status

**DOI:** 10.1007/s00701-025-06616-2

**Published:** 2025-07-22

**Authors:** Yash Akkara, Rachel Fernandes, Ping Ping Wang, Aron Abraham, Hamza Bhat, Joe M. Das, Ramesh Nair, Nigel Mendoza

**Affiliations:** 1 Imperial College School of Medicine, London, UK; 2 Imperial College Healthcare NHS Trust, London, UK

**Keywords:** Frailty, Meningioma, Surgery, Elderly, Tumour, Neurosurgery

## Abstract

**Introduction:**

Meningioma resection constitutes a substantial portion of neurosurgical practice, with a variety of patient-factors playing a role in outcomes. This study aims to assess the role of frailty in elderly patients undergoing resection of primary intracranial meningiomas.

**Methods:**

This is a retrospective study of patients undergoing resection for primary intracranial meningiomas at our institution between 2004–2024. Patients were ≥ 65 years, and were required to have ≥ 1 year of follow-up. The modified frailty index (mFI) was used to identify degree of frailty, categorising patients into the non-frail (NF, mFI = 0), pre-frail (PF, mFI = 1/2), and frail (F, mFI ≥ 3) groups. The Shapiro–Wilk test, paired T-test, and ANOVA were used to identify statistical significance. Propensity-score matching (PSM) was performed based on a multinomial logistic regression model and 1:1 nearest-neighbour matching algorithm. Kaplan–Meier analysis using Log-rank tests were used to compare overall (OS) and progression-free (PFS) survival.

**Results:**

362 patients were included, with 69, 207, and 86 patients in the NF, PF, and F groups respectively. Preoperatively, the F group was found to have a significantly higher age vs. the NF group (76.17 vs. 71.57, p = 0.0002), alongside reporting a significantly lower median KPS vs. the PF and NF groups (70 vs. 80, p < 0.0001) respectively. The PF group was found to have a significantly lower duration of surgery (309 min, p = 0.0048) vs. the F group, which reported significantly higher mean length-of-stay (17.68 days, p < 0.0001), higher frequencies of medical complications (11.6%, p = 0.0317), and lower postoperative KPS (70, p < 0.0001) vs. other groups. Despite PSM, the F group was found to have significantly reduced median OS (p = 0.0106) and PFS (p = 0.0101) compared to other groups.

**Conclusion:**

Frailty was significantly associated with higher length-of-stay, frequent medical complications, and worse OS and PFS following surgery vs. other groups. Frailty is hence a crucial consideration in preoperative planning for elderly populations undergoing meningioma resection.

## Introduction

Meningiomas represent the most common primary intracranial tumours in adults, accounting for approximately 37% of all primary central nervous system tumours [24]. These tumours are typically benign and slow growing; although, a small subset can be atypical or malignant, posing significant clinical challenges. Their incidence rises significantly as age increases, disproportionately affecting the elderly [1]. Surgery is the primary modality of treatment, with radiation therapy and, less frequently, chemotherapy used as adjuncts [2].

Due to the significantly higher incidence of meningiomas in the elderly, it is essential to consider the role of frailty on outcomes following resection [1]. Frailty is a multidimensional condition broadly characterised by “decreased physiological reserve” and increased vulnerability to stressors. The mechanisms underlying frailty emerge from interconnected physiological and molecular processes that impair homeostasis and stress responses. It is characterised by adverse health outcomes such as weakness, weight loss and low activity [9]. Prevalent in ageing populations, frailty has become increasingly pertinent in neurosurgical contexts, with research focussing more on its role on outcomes across neurosurgery.

Across several domains of medicine, frailty is a major determinant of outcomes and mortality [9]. Studies have shown a significant disparity between healthy and frail patients within the context of surgery, with frail patients losing approximately 100 days of home time per year as a result of surgical intervention [26]. Frailty is also associated with worse short and long-term postoperative outcomes, regardless of operative stress, being up to 3 times more likely than non-frail patients[12], alongside a nearly fourfold increase in mortality [26].

Some studies have attempted to characterize the role of frailty on outcomes following surgery for meningiomas, generally highlighting its negative prognostic role on complications and outcomes across general populations [7, 29]. However, to our knowledge, there are no studies specifically identifying outcomes in the elderly — the population most strongly affected by increased frailty. Moreover, few studies have attempted to isolate the role of frailty itself, through elimination of baseline variables such as age and functional status that can substantially confound results regarding operative outcomes. While studies by Elsamadicy et al. [7] and Theriault et al. [29] have explored the link between frailty and outcomes following meningioma resection, these studies include a general meningioma population, while not controlling for the confounding effect of baseline functional deficits. These studies also span a study interval of 3–5 years, necessitating more robust and longitudinal analyses. Given the crucial prevalence of frailty in the elderly, alongside the need to isolate the role of frailty on the postoperative recovery process to effectively identify the extent to which it affects outcomes, our study employed a propensity-score matched analysis within an elderly cohort undergoing resection for meningiomas, with the aim to investigate its prognostic value on a variety of surgical outcomes in a longitudinal manner.

## Methods

### Study design

This is a retrospective study of elderly patients undergoing resections of histologically confirmed meningiomas between 2004 and 2024 at the Imperial College Healthcare NHS Trust (ICHNT) in London. All patient data was obtained from the Trust’s electronic medical record (EMR) system through a formal audit request (Audit Identifier: NSG_06).

### Participants

Inclusion was limited to patients ≥ 65 years of age with a histologically confirmed diagnosis of a meningioma who underwent resection at our institution. All patients were also required to have complete follow-up data available up to 1-year following resection on the EMR system, and to have complete clinical profiles and notes. Patients with incomplete, inconclusive, or withdrawn data from charts were excluded from the study.

### Outcomes

A variety of outcomes were extracted retrospectively from patient records. These included demographic characteristics (gender, age, deprivation status), tumour characteristics (size, location, grade, consistency), clinical characteristics (preoperative Karnofsky performance scale/KPS, modified frailty index/mFI), and postoperative outcomes (duration of surgery, length-of-stay, KPS at discharge, KPS at 6-months postoperatively, extent of resection, complications, reoperations, progression-free survival, and overall survival). The modified frailty index (mFI) was used as the primary method of characterizing frailty status [8]. This is a score out of 11, with each of the following parameters worth 1-point (Table 1). Complications were classified into “medical” or “surgical” complications using the Landriel-Ibañez classification system [18], which establishes a neurosurgery-specific criteria for differentiating medical complications as those not directly related to surgical technique (including pneumonias, acute kidney injury, thromboembolism, etc.), and surgical complications as those which stem directly from surgical technique (CSF leak, wound infection, haematomas, vascular injuries, etc.). All authors were blinded to mFI status prior to documenting the patients’ surgical history (only reviewing the relevant surgical and outpatient notes corresponding to their admission), with mFI calculated by a separate author based on the patient’s longitudinal medical history. Patients with missing data for the aforementioned outcomes, particularly for patients prior to 2013 with data not on the EMR system, were excluded from the analysis. Due to the consistent documentation of all conditions within a patient’s medical history across the EMR system that is performed on admission, all data regarding mFI status were obtained directly from their records. Any patients with incomplete or ambiguous documentation were excluded from the study. Data for patients operated on prior to 2013 were identified using a formal search to the ICHNT database, using a combination of physical reports and stored imaging/operative notes on a secure server.

**Table 1:** Modified frailty index (*mFI-11) criteria*

Criterion	Weightage
Diabetes Mellitus	1 point
Congestive Heart Failure	1 point
Hypertension (requiring medication)	1 point
Stroke or Transient Ischemic Attack	1 point
Functional Status (Independent vs. Dependent)	1 point
Myocardial Infarction	1 point
Vascular Disease	1 point
Neurological Deficit	1 point
COPD or Pneumonia	1 point
Previous Percutaneous Coronary Intervention, Cardiac Surgery, or Angina	1 point
Impaired Sensorium	1 point

### Classification and comparison

Based on the mFI score, patients were characterized into 3 groups: non-frail (mFI = 0), pre-frail (mFI = 1/2), and frail (mFI ≥ 3). All postoperative outcomes were analysed across the 3 groups to identify the role of frailty on statistically significant differences in outcomes. All classification of frailty was performed following data-collection by a separate author to mitigate potential bias. Any discrepancies in classification were resolved through consensus. To prevent confounding due to baseline characteristics that may inadvertently skew outcomes (such as baseline differences in functional status, tumour characteristics, etc.), propensity-score matching was performed on all 3 groups with respect to all significantly different baseline variables. This generated 3 equally sized groups, matched to optimize baseline congruence with the group with the smallest sample.

### Statistical analysis

The Shapiro–Wilk test, paired T-test, and ANOVA test were used to identify statistically significant differences across patient groups, both for baseline and postoperative characteristics. Tukey test analyses and box-and-whisker plots were used to depict descriptive statistics of patient groups, with outliers depicted with their specific data points. Propensity-score matching (PSM) was performed based on a multinomial logistic regression model and a 1:1 nearest-neighbour matching algorithm. Standardized mean differences (SMDs) were calculated before and after matching to assess balance, with an |SMD|< 0.1 considered indicative of adequate balance. Kaplan–Meier curves were generated to document progression-free and overall survival across the 3 groups following resection, with the Log-rank (Mantel-Cox) test used to identify statistically significant differences in survival trajectories across the groups. To account for differences in follow-up across patients, patient data was censored at the time of their latest follow-up within the KM analysis to ensure accurate depiction of survival trends.

## Results

### Included participants

A total of 2318 resections of histologically confirmed meningiomas were performed at our centre between 2000 and 2024. Between 2000 and 2013, we were only able to retrieve sufficient data for 40 patients aged 65 years or older, owing to patient data being stored outside the EMR system. Following 2013, all patient records were transferred to the EMR system, with a total of 1391 resections carried out between 2013 and 2024. Of these resections, 541 were carried out on individuals aged 65 years and older. Within this group of patients, 159 did not have sufficient follow-up outcome and survival data, while 34 were operated on in 2024, and hence did not have 1-year of survival data. Finally, 26 patients did not have/withdrew their records on our EMR system, and hence could not be included.

This left 362 patients who formed the final cohort included within the study, consisting of 130 men and 232 women, with a mean age of 74.18 (7.18) years. All patients were aged 65 years and older, and were divided into 3 groups: non-frail (NF, mFI 0), pre-frail (PF, mFI 1 and 2), and frail (F, mFI 3 and above). There were 69, 207, and 86 patients in the NF, PF, and F groups respectively. Mean follow-up was 57.1 months. Participant demographic characteristics are further described in Table 2.

**Table 2:** Baseline demographic factors of included patients

Variable		Value
Gender	Male	130 (35.9%)
	Female	232 (64.1%)
Age (mean, SD, years)		74.18 (7.18)
Surgery duration (mean, SD, minutes)		375.27 (148.8)
Length of Stay (median, IQR, days)		8 (5–15)
Deprivation Decile (median, IQR)		5 (4–7)
Tumour Size (mean, SD, mm)		41.20 (18.84)
Tumour Location	Convexity	104 (28.7%)
	Parasagittal	91 (25.1%)
	Sphenoid Wing	64 (17.7%)
	Olfactory Groove	42 (11.6%)
	Cerebellopontine Angle	19 (5.2%)
	Suprasellar	13 (3.6%)
	Clinoid	8 (2.2%)
	Other	21 (5.8%)
Karnofsky Performance Status (Pre-op)		80 (70–90)
Karnofsky Performance Status (Discharge)		80 (70–90)
Karnofsky Performance Status (6-months)		80 (70–90)
Frailty Status (Modified Frailty Index/mFI)	Non-Frail (mFI = 0)	69 (19.1%)
	Pre-Frail (1 ≤ mFI ≤ 2)	207 (57.2%)
	Frail (mFI ≥ 3)	86 (23.8%)
Extent of Resection	Subtotal	129 (35.6%)
	Total	205 (64.4%)
Medical Complications	No	344 (95.0%)
	Yes	18 (5.0%)
Surgical Complications	No	292 (80.7%)
	Yes	70 (19.3%)
Reoperations	No	317 (87.6%)
	Yes	45 (12.4%)
Post-discharge destination	Home	241 (66.6%)
	Rehabilitation Facility	34 (9.4%)
	Another ward/hospital	60 (16.6%)
	Hospice/End-of-Life Care	13 (3.6%)
	Unclear	14 (3.9%)

### Baseline differences across groups and matching

**Fig. 1:**
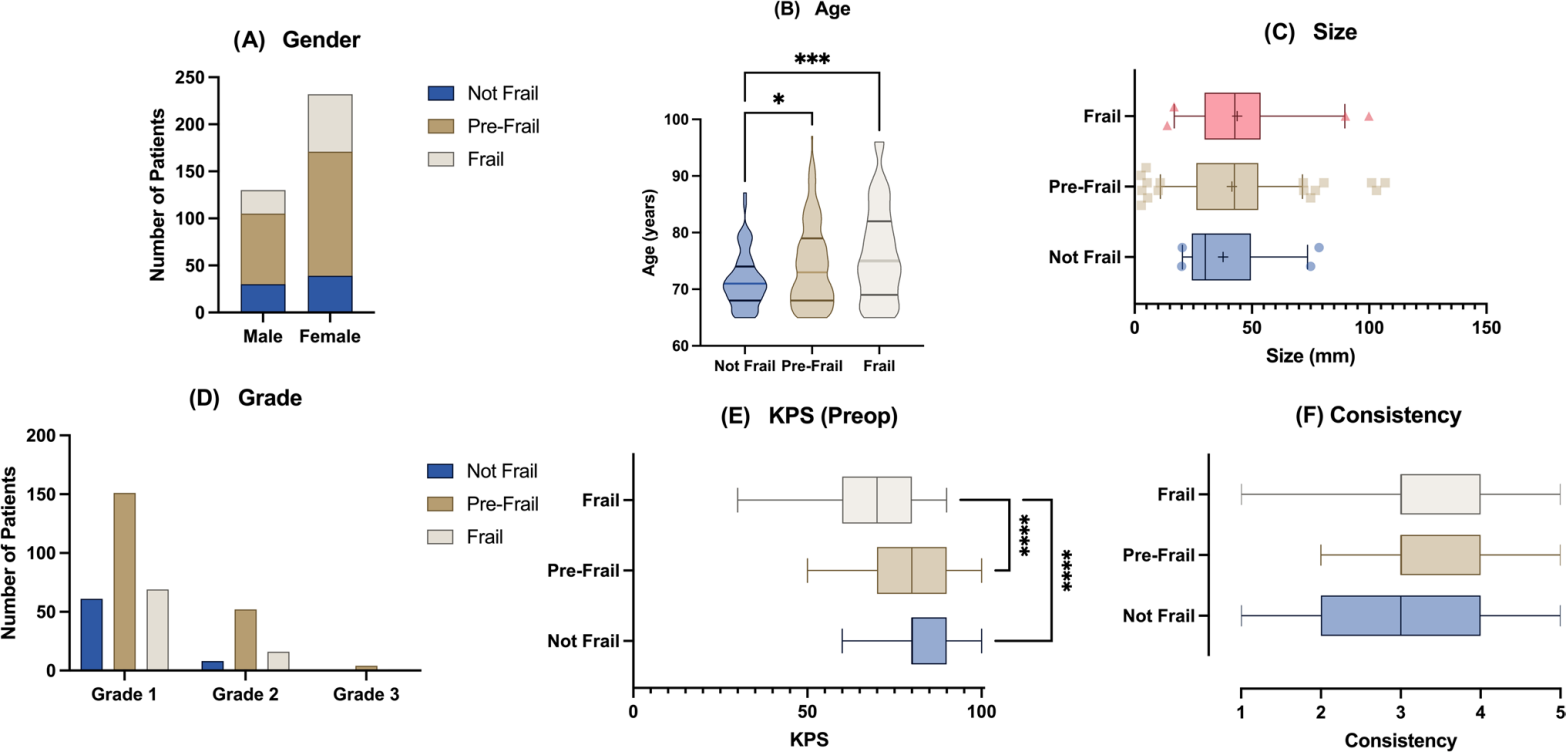
A-F: Differences in baseline characterises including gender (A), age (B), tumour size (C), tumour grade (D), preoperative Karnofsky Performance Status (KPS) (E), and tumour consistency (F) across the 3 groups

Participant baseline demographic and clinical characteristics were compared across the 3 groups to identify significant differences that may affect comparisons of outcomes. No significant difference was found in the proportion of male and female patients across the 3 groups (Fig. 1A). The NF group reported significantly lower mean age vs. the PF (71.57 vs. 74.23, p = 0.0186) and F groups (71.57 vs. 76.17, p = 0.0002) respectively (Fig. 1B). No significant differences were observed in mean tumour size across the 3 groups, with an average of 37.74 mm, 41.43 mm, and 43.71 mm in the NF, PF, and F groups respectively (Fig. 1C). Similarly, no significant differences were observed in tumour grade across the 3 groups, with all groups majorly consisting of grade-1 tumours (Fig. 1D). Significant differences were observed across all 3 groups with regards to preoperative KPS (Fig. 1E). The F group was found to have a significantly lower median preoperative KPS vs. the NF (70 vs. 80, p < 0.0001) and PF (70 vs. 80, p < 0.0001) groups respectively. Finally, no significant differences were found in tumour consistency across the 3 groups (Fig. 1F).

Based on these findings, propensity-score matching was performed across all 3 groups, with priority of matching given to KPS, followed by age, and gender based on the limiting group (NF, n = 69). As such, within the propensity-score matched analysis, all groups had a sample of 69 participants, with a total sample of 207 patients. The propensity-score matching was highly successful for KPS, age, and gender, achieving |SMD|< 0.1 for all 9 comparisons. This provided significantly more comparable datasets compared to the initial sample, which only achieved |SMD|< 0.1 for 4/9 comparisons (Table 3).

**Table 3:**
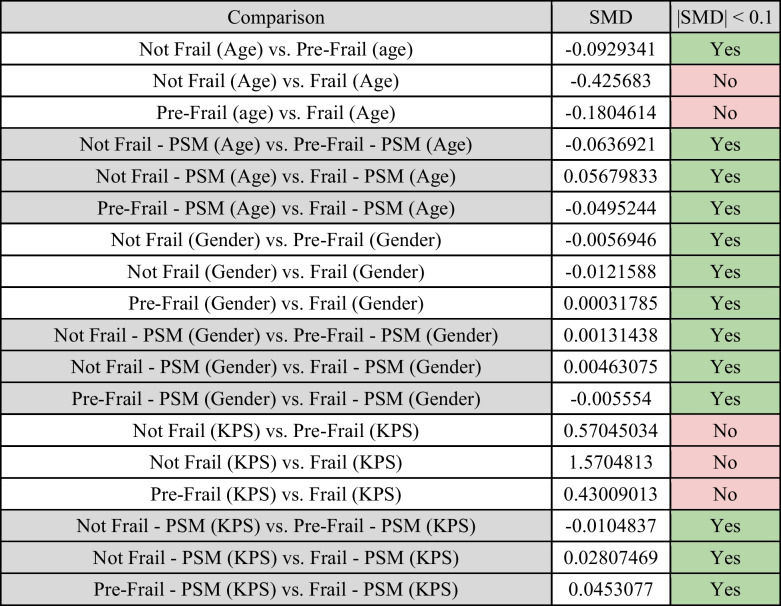
Standardised mean differences (SMD) of all non-propensity score matched (non-PSM) and propensity score matched (PSM) comparisons across the 3 groups

### Postoperative outcomes

**Fig. 2:**
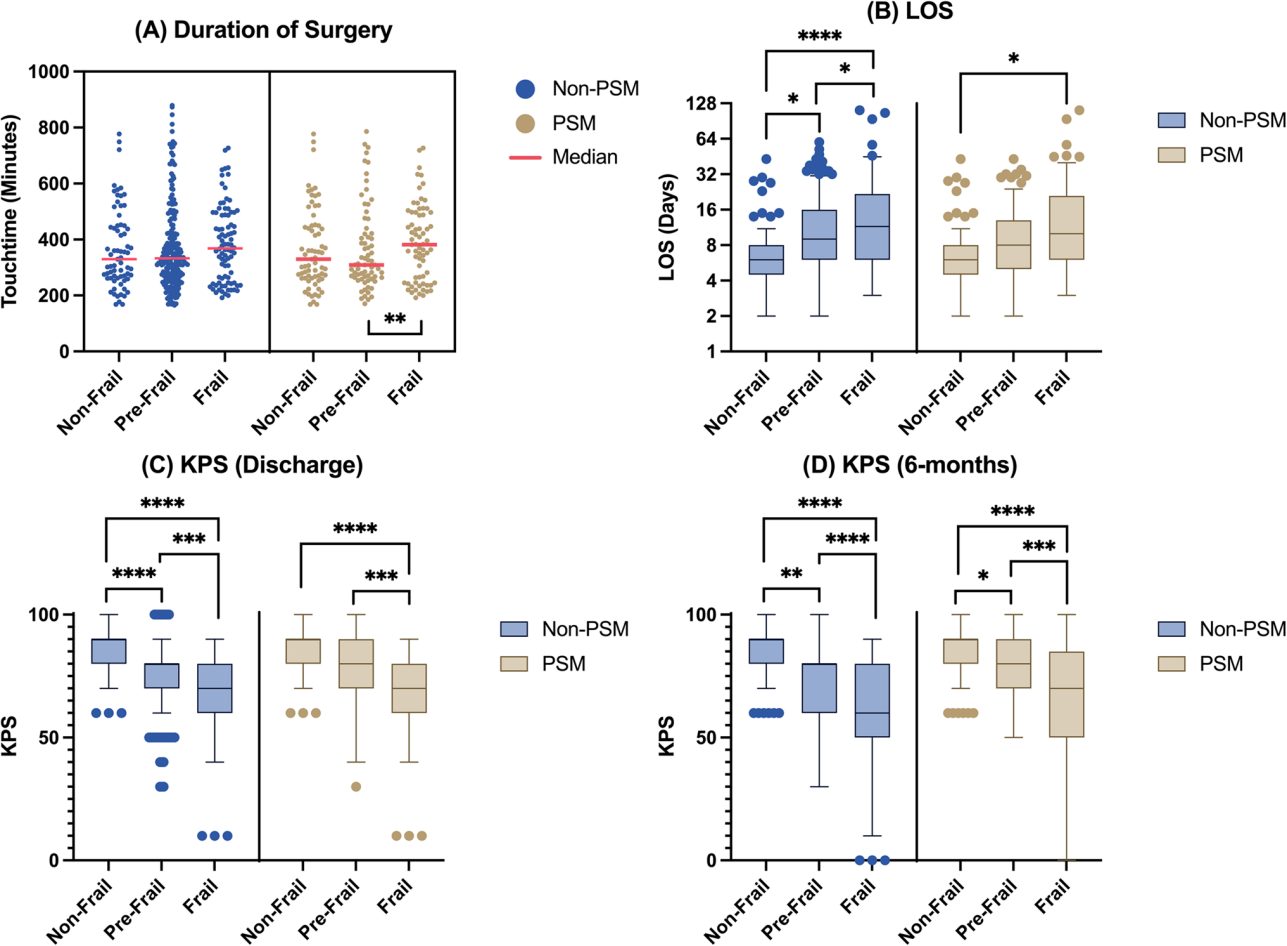
A-D: Postoperative differences in duration of surgery (A), length-of-stay (B), Karnofsky Performance Status (KPS) at discharge (C), and Karnofsky Performance Status (KPS) at 6-months (D) across the groups, prior to and following propensity-score matching (PSM)

Prior to PSM, no significant differences were found in median duration of surgery across all 3 groups. Following PSM, the PF group was found to have a significantly median lower duration vs. the F (309 vs. 382 min, p = 0.0081) group (Fig. 2A). To account for the differences in operative approaches across groups, we compared differences in the frequency of the 5 most frequent tumour locations (convexity, parasagittal, sphenoid-wing, olfactory groove, cerebellopontine angle), finding no significant differences in tumour distributions across the 3 groups, both prior to (F = 1.209, p = 0.303) and following PSM (F = 1.858, p = 0.010). On the other hand, prior to PSM, the F group was found to have a significantly higher mean LOS vs. the NF (17.68 vs. 8.01 days, p < 0.0001) and PF groups (17.68 vs. 12.73 days, p = 0.0134). Similarly, the NF group also showed a significantly lower mean LOS vs. the PF group (8.01 vs. 12.73, p = 0.0101). Following PSM, only the NF group reported a significantly lower LOS vs. the F group (8.01 vs. 17.25, p = 0.0425), with no other significant differences (Fig. 2B*)*. KPS at discharge was found to be significantly different across the 3 groups before and after PSM. Prior to PSM, the F group was found to have a significantly lower median KPS vs the NF (70 vs. 90, p < 0.0001) and PF groups (70 vs. 80, p = 0.0004). The PF group was also found to have a significantly lower mean postoperative KPS vs. the NF group (80 vs. 90, p < 0.0001). Following PSM, similar relationships were observed between the F vs. NF groups (70 vs. 90, p < 0.0001) and F vs. PF (70 vs. 80, p = 0.0005) groups. No significant differences were observed between the PF and NF groups (Fig. 2C*)*. Finally, we compared KPS at 6-months postoperatively across the groups, with the F group continuing to report significantly worse KPS vs. both the NF (60 vs. 90, p < 0.0001) and PF (60 vs. 80, p < 0.0001) groups prior to matching. Following matching, a similar trend was observed, with the F group reporting significantly worse KPS vs. the NF (70 vs. 90, p < 0.0001) and PF (70 vs. 80, p = 0.0004) groups respectively (Fig. 2D).

**Fig. 3:**
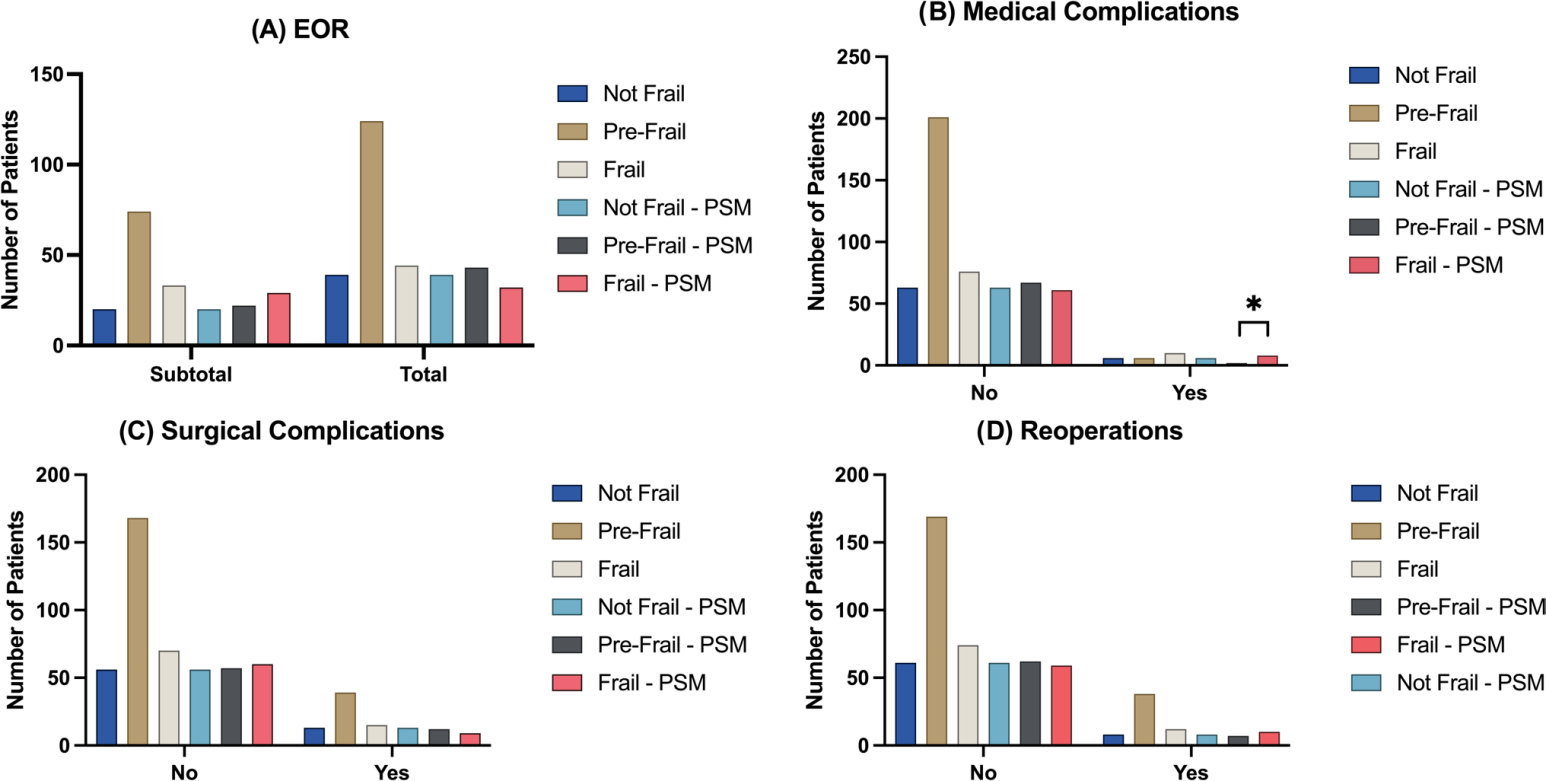
A-D: Postoperative differences in extent-of-resection (EOR) (A), medical complications (B), surgical complications (C), and reoperations (D) across the groups, with and without propensity-score matching (PSM)

With and without PSM, no significant differences were found in extent-of-resection across the 3 groups (Fig. 3A*)*. Prior to PSM, no significant differences were observed in the frequency of medical complications across the 3 groups. However, following PSM, the F group was found to have a significantly higher frequency of medical complications vs. the PF (11.6% vs. 2.9%, p = 0.0317) group (Fig. 3B). On the other hand, no significant differences were observed across the 3 groups with regards to surgical complications (Fig. 3C*)* or the frequency of reoperations, both prior to and following propensity-score matching *(*Fig. 3D).

### Postoperative survival

**Fig. 4:**
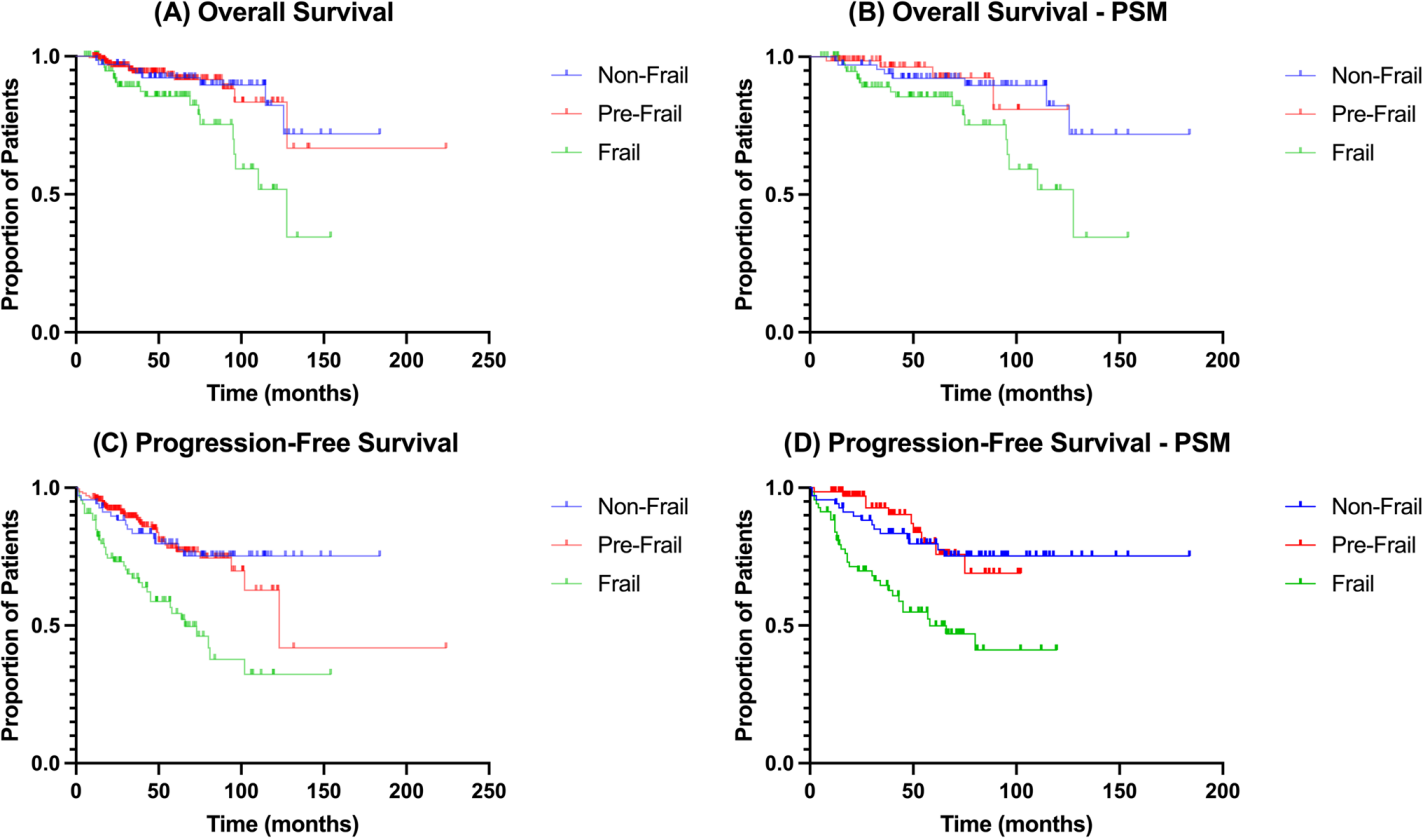
A-D: KM differences in overall survival (A), propensity-score matched (PSM) overall survival (B), progression-free survival (C), and propensity-score matched (PSM) progression-free survival (D) across the 3 groups

Prior to PSM, the F group (median survival = 128 months) was found to have a significantly worse overall survival vs. the NF (p = 0.0094) and PF (p = 0.0081) groups (Fig. 4A). Following PSM, the F group (median survival = 110 months) continued to report significantly lower overall survival vs. both the NF (p = 0.0187) and PF (p = 0.0106) groups (Fig. 4B). Similarly, prior to PSM (Fig. 4C), the NF group was found to have significantly higher progression-free survival vs. the PF (median PFS = 123 months, p = 0.0007) and F (median PFS = 66 months, p < 0.0001) groups. The PF group was also found to have a significantly higher progression-free survival vs the F group (p = 0.0204). However, following PSM, no significant difference was observed between the NF and PF groups in progression-free survival. The F group continued to show worse progression-free survival (median PFS = 58 months) vs. the NF (p = 0.0101) and PF (p = 0.0126) groups (Fig. 4D).

## Discussion

This study presents novel findings regarding the impact of frailty on postoperative outcomes in elderly patients undergoing resection of meningiomas. We demonstrated that frailty status, as assessed by the modified frailty index (mFI), significantly influences inpatient recovery, the risk of medical complications and long-term survival, independent of baseline age and functional status. These findings provide valuable insight for clinicians in preoperative risk evaluation and patient counselling, emphasising the need for frailty assessment in surgical decision-making and postoperative care planning.

Age was found to be significantly different between the NF and F groups, with frailer patients being older on average. This finding aligns with existing literature, which associates advancing age with increased frailty due to progressive physiological reserve depletion, accumulation of comorbidities and overall functional decline [6, 13]. Preoperative KPS scores were also significantly lower in frail patients, reinforcing the established link between frailty and baseline functional impairment. Tumour-related characteristics such as size, grade, and consistency did not differ significantly across groups, suggesting frailty itself does directly influence tumour biology. However, it is important to note that although both age and KPS at baseline were significantly different across groups, these may not necessarily represent clinically meaningful differences. Although factors such as increased age and reduced KPS have been shown to be linked with increased frailty, the groups generally fell within ± 10% of each-other, suggesting that other factors/comorbidities (such as those in the mFI) are also highly relevant. Nevertheless, matching for age, baseline KPS, and sex ensures preoperative functional deficits are controlled for, allowing differences in postoperative function and outcomes to be isolated as much as possible to frailty itself.

Following PSM, the PF group demonstrated a significantly lower median duration of surgery compared to the F group, despite no significant differences in the distribution of tumour locations across the three groups. This difference may be explained when considering anaesthesia, as frail patients often require more cautious anaesthesia management [20]. Furthermore, frail patients have been shown to have an increased likelihood of intraoperative challenges due to factors such as vascular and tissue fragility, potentially contributing to prolonged procedural durations [3]. The frail group was also found to have a significantly higher length-of-stay (LOS) compa to other groups, with this finding persisting in comparison to the NF group following PSM. This result aligns with previous studies; with research finding that frailty is associated with a prolonged duration of stay on univariate and multivariate analysis across a variety of surgical procedures [22]. KPS at discharge remained significantly different across the three groups before and after PSM. Despite accounting for baseline differences in KPS, frail patients continued to report substantially worse functional outcomes on discharge relative to both PF and NF groups demonstrating the direct role frailty on functional recovery. Similarly, the PF group also reported significantly lower postoperative scores vs. the non-frail group (including following PSM), suggesting the relatively milder but still substantial effect of comorbidities and frailty on functional outcomes. At 6-months following surgery, these differences were more apparent, with the F group reporting significantly lower median KPS vs. discharge (60 vs. 70). Although this deterioration was no longer observed following PSM, these findings suggest that frail patients experience greater rates of physiological decline, often following events of prolonged inactivity such as surgery and inpatient stays. These can be attributed to the reduced physiological reserves and higher complication rates known to be associated with frailty in the literature, leading to worse outcomes and limited recovery potential [1, 9, 25].

The F group was linked with significantly higher frequencies of medical complications when compared with the PF group, reinforcing the idea that frailty predisposes patients to worse postoperative outcomes. The increased frequency of medical complications in frail patients can be attributed to their lower physiological reserve and factors such as immunosenescence. Reduced physiological reserve results in diminished recovery capacity following surgical stress [4]. Additionally, a weakened immune system delays tissue regeneration and impairs wound healing, increasing susceptibility to postoperative infections and, in severe cases, sepsis. Prolonged immobility, due to frail patients taking longer to mobilise postoperatively, also increases the risk of deep vein thrombosis, which can lead to further complications such as pulmonary embolisms [28, 30]. However, no significant differences were noted in surgical complications, reoperations or extent of resection across the groups suggesting no association between frailty and intraoperative outcomes. This is a highly relevant finding reinforcing the distinction between technical and physiological factors as predictors of operative risk. Since factors such as surgical complications and reoperations are often more dependent on the surgical procedure itself (including extent-of-resection, infection control, etc.) as opposed to physiological factors including frailty, they may not vary significantly across groups [10]. This is further supported by similar differences in surgical duration across the 3 groups, suggesting consistency in surgical and operative technique in the peri-operative period. This can be explained by factors such as surgical complications, EOR, and duration being driven primarily by surgical expertise, technique, and planning, which often remain consistent within meningioma resections, regardless of physiological patient factors [17]. This finding also highlights the need for greater involvement of surgeons and clinicians in the postoperative care of patients with increased frailty, as changes to intraoperative techniques may be unlikely to result in significant improvements in outcomes in comparison to inpatient support and medical management.

KM analysis found overall survival (OS) was significantly lower in the F group compared with PF and NF groups, before and after PSM, highlighting that frailty independently contributes to worse long-term survival irrespective of age, gender or baseline functional status. This can partially be attributed to the increased frequency of medical complications and multimorbidity associated with frailty, both of which exacerbate long-term outcomes. This finding is supported by literature on the impact of frailty on patient outcomes, which demonstrates that frailty scores are associated with 30-day mortality on univariate and multivariate analyses [22]. Similarly, progression-free survival (PFS) was found to be significantly lower in the F group relative to the NF groups before PSM, and between the F group and both NF and PF groups after PSM. This suggests frail patients are more likely to experience earlier disease recurrence or progression. The reduced tolerance of frail patients to adjuvant therapies such as radiotherapy and chemotherapy, along with immunosenescence and weakened immune function, may impair the body’s ability to eliminate residual cancer cells postoperatively [14, 21]. Moreover, the lower PFS in the PF group, which was found to be non-significant following PSM, highlights that frailty itself was not the primary driver of these discrepancies, but rather a combination of frailty and confounding variables. As such, the findings of this study highlight how individuals in the PF group, while at risk of increased frailty, can still report satisfactory OS and PFS outcomes with early identification and intervention, primarily through preventing further deterioration and increased frailty.

The study presents novel findings about the impact of frailty on patient outcomes following meningioma resection: frail patients had a higher risk of medical complications, a decrease in both progression-free and overall survival and a lower median postoperative KPS. It also highlighted that, although pre-frail individuals often reported significantly lower postoperative KPS scores compared to the non-frail group, early identification and intervention in these patients could mitigate the impact of frailty, resulting in comparable OS and PFS outcomes. These findings reinforce the overall detriment frailty brings in a variety of neurosurgical procedures, which has been described in resections of other primary brain tumours [27], clipping of aneurysms [15], or intracerebral haemorrhage [19]. This study further illustrates the extent to which frailty resonates throughout neurosurgical populations, emphasising the need for preoperative frailty assessments by clinicians. This is also the first study to restrict the sample to elderly patients (≥ 65 years of age), providing significantly more depth into the analysis of outcomes in the elderly. Our large sample of patients, alongside the use of PSM, enhanced the reliability of the comparisons made between groups. Additionally, the longitudinal follow-up period averaged around four years, providing valuable long-term outcome data. This is especially of note with our minimum inclusion of ≥ 1-year of follow-up following surgery, ensuring no data was lost to follow-up within the postoperative recovery period, allowing the direct identification of factors such as complications, recurrence, and KPS that permits clinicians to characterise the general recovery process for their elderly patients. The use of stratified analysis allowed for identification of differences in various outcomes across the groups, providing a more detailed understanding of the impact of frailty on patient outcomes. Through our findings, we aim to equip clinicians with the means of identifying frail patients at risk of worse outcomes, allowing early intervention. These include prehabilitation programs targeting nutrition, physical function, and medical optimization, which have been shown to improve surgical resilience [11]. The use of multidisciplinary care involving geriatricians and rehabilitation teams has also demonstrated improved postoperative outcomes in frail populations [23], while enhanced recovery protocols (including early mobilization and complication prevention) have been shown to reduce morbidity and length of stay [16]. We hope our findings can allow increased deployment of these measures in at-risk patients, particularly for those within the pre-frail category where early intervention may be optimally effective at maintaining progression-free and overall survival.

### Limitations

This study is limited by its retrospective design, which may introduce selection bias, as data collection was limited to available records rather than a randomised or prospective approach. A key limitation of the study is the lack of standardised data storage at our institution, leading to the exclusion of a large number of patients prior to 2013. This was due to a switch in data-storage modality in 2013 to the EMR system, with only a few patients (n = 40) having comparable clinical data who were operated on between 2004–2013. This also reduced the extent to which we were able to study longitudinal trends across patients, with only a fraction of patients having follow-up data ≥ 10 years following resection. Another limitation of this study is the potential for loss to follow-up. While we included only patients with at least one year of documented clinical and radiological follow-up to minimise this, there remains a possibility of unrecorded care received outside our system, which may have led to underestimation of events such as recurrence or delayed complications. While our institution maintains a comprehensive and integrated electronic health record system, the retrospective design inherently limits our ability to capture outcomes occurring outside this network. Additionally, key variables such as the mFI, KPS and tumour consistency were estimated based on retrospective data, which could not be confirmed prospectively, leading to the potential for information bias. While the mFI is highly amenable to retrospective data collection, owing to its focus on prior health conditions (as opposed to variables such as daily functional status or activities of daily living which often require prospective confirmation), there remains potential for misclassification. To reduce this risk, frailty components were abstracted from detailed clinical notes and standardized data fields, with discrepancies resolved through consensus. Future studies may benefit from the use of other validated frailty-assessment systems, notably the clinical frailty scale [5], which may be more effective in measuring day-to-day frailty, compared to the mFI which relies on the individual’s medical history. Moreover, through our inclusion criteria, a variety of patients with valuable data were excluded from the analysis, including those under the age of 65, patients with incomplete surgical data, and patients with inadequate follow-up. We were also unable to perform sensitivity analyses in this study due to the inherent limitations of its retrospective design and the constraints in controlling for unmeasured confounders. The primary focus was on evaluating associations rather than establishing causality, which reduced the feasibility of applying sensitivity checks within the existing dataset. In future studies, particularly those with prospective or multicentre designs, sensitivity analyses could be incorporated through stratified subgroup evaluations or varying matching parameters in propensity-score models to assess the robustness and generalizability of findings across different patient populations and clinical scenarios. Finally, changes in surgical techniques and methods over time were not accounted for, which could have influenced patient outcomes across our follow-up period.

This study also has several limitations in the data we collected. First, a significant amount of missing data led to the exclusion of patients, potentially impacting the generalisability of the findings. Additionally, there was considerable variation in follow-up duration, with some patients having significantly shorter follow-up periods than others, which may have affected the assessment of long-term outcomes. The study also could not establish causality due to its observational nature. We also had a relatively small sample within the NF group (n = 69). This is likely due to the restriction to patients who were ≥ 65 years of age, increasing the likelihood of comorbidities that reduces the proportion of patients scoring ‘0’ on the mFI. Thus, to allow for equal comparisons within the PSM analysis, 69 patients were chosen from the 2 other groups (via the 1:1 nearest neighbour method), reducing our overall statistical power. This, coupled with the necessary exclusion of patients with missing EMR data, may limit the external validity of our findings, and reduces the study’s power to detect other relevant roles of frailty on outcomes. Further work in prospective studies with larger cohorts may be required to better understand the effect of frailty on outcomes following meningioma resection. Finally, although a variety of statistically significant differences were observed across the groups, the clinical significance of these variables may not be as apparent, especially with factors such as marginal differences in length-of-stay and postoperative median KPS. Therefore, care should be taken in the interpretation and application of our results, as these differences may not be apparent on a case-by-case basis, and hence should not be used as a method of inferring frailty status. Instead, a formal assessment of frailty using tools such as the mFI or clinical frailty scale is still required for an accurate estimate. As such, the results require further validation in larger, more controlled cohorts to further ensure the effect of frailty is isolated as much as possible.

## Conclusion

This study highlights the significant impact of frailty on postoperative outcomes in elderly patients undergoing meningioma resection. Frailty was associated with a higher risk of medical complications, prolonged hospital stays, worse functional recovery, and reduced long-term survival. It has also highlighted that although individuals who are “pre-frail” developed worse postoperative functional outcomes, the prevention of further frailty through early identification can permit satisfactory overall and progression-free survival. The use of PSM enhanced comparability across groups, confirming that frailty itself, rather than other confounding variables, has a key role in these outcomes. These findings underscore the importance of incorporating frailty assessments into preoperative planning to improve surgical decision-making and post-operative care, to ultimately improve patient outcomes.

## Data Availability

No datasets were generated or analysed during the current study.

## Declarations

### Institutional approval

This is a registered and approved audit of patients treated at our institution through resection of meningiomas, approved by the neurosurgical audit committee. The approved audit identifier in the ICHNT database is **NSG_06**.

### Submission declaration

This work has not been published, and is not currently under consideration elsewhere.
